# An unusual case of Pisa syndrome secondary to anti-IgLon 5 disease

**DOI:** 10.1016/j.prdoa.2026.100428

**Published:** 2026-02-19

**Authors:** Sindhu V. Nambiar, Thomas Mathew, Vishal Chandra Sharma, Arjun Jayaprakash, Shagun Bhardwaj, Anita Mahadevanan

**Affiliations:** a Department of Neurology, St. John’s Medical College Hospital, Sarjapura Road, Bengaluru, Karnataka 560034, India; b Department of Pathology, National Institute of Mental Health and Neurological Sciences (NIMHANS), Bangalore, India

**Keywords:** Pisa syndrome, AntiIglon 5 disease, Head drop, Orolingual dyskinesias, Taupathy

## Abstract

Pisa syndrome, characterized by lateral flexion of the trunk, is most often associated with neuroleptic use or neurodegenerative disorders like Parkinson’s disease. Anti-IgLON5 disease is a rare neuroimmunological condition with overlapping features of autoimmunity and tauopathy, manifesting with sleep and cognitive disturbances, bulbar symptoms, and movement disorders. We report a 50-year-old man who presented with progressive lateral trunk flexion, head drop, dysarthria, and choreiform movements. The classic features of antiIgLon 5 disease including sleep and cognitive disturbances were absent. Serum testing confirmed strong positivity for anti-IgLON5 antibodies. Treatment with rituximab led to marked clinical improvement, with amelioration of symptoms at 24 months. This case illustrates an uncommon presentation of anti-IgLON5 disease as Pisa syndrome. Recognition of this association is important, as early immunotherapy can significantly improve outcomes in an otherwise progressive and disabling condition.

## 1 Introduction

PISA syndrome (PS), also known as pleurothotonus, is characterized by reversible lateral bending of the trunk, resulting in leaning to one side. Common causes of PS include the use of antipsychotic drugs and neurodegenerative disorders such as Parkinsonism. Two distinct pathophysiological mechanisms have been proposed for PS: a central mechanism involving dysfunction of the basal ganglia networks, and a peripheral mechanism related to spinal osteoarticular changes and paraspinal myopathy. We report a case of PS secondary to antiIgLON5 antibody disease. We also highlight the favorable response to rituximab treatment. Our case is unique in that he did not have the characteristic features of anti IgLon 5 disease like sleep and cognitive disturbances even on thorough evaluation. Also , he responded very well to rituximab with no progression of symptoms even at 2 years followup.

## 2 Case report

**Fig. 1:**
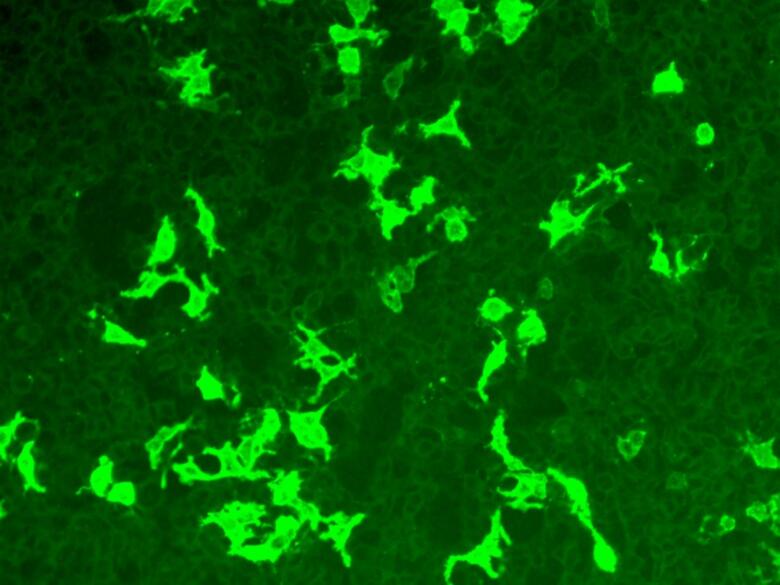
Strong immunofluorescence in a cell-based assay using indirect immunofluorescence on transfected cells in serum.

A 50-year-old man presented with leaning to his left side, a forward drooping of the head, difficulty in swallowing and speaking, and instability while walking for two months duration. The disease was insidious in onset and progressive in nature. On examination, he had excessive sweating over the face and trunk. He had a lateral bend of the trunk to the left side (supplementary video 1) and a neck drop (supplementary video 2) , a hypophonic voice, and slow and slurred speech. He was conscious and oriented and his higher cognitive functions including attention and memory were normal. Cranial nerve examination was normal except for broken pursuit eye movements. He had perioral and lingual dyskinesia (supplementary video 3) . He also had choreiform movements of all four limbs, more prominent on the left side. Power was normal in limb and neck muscles . Deep tendon reflexes were normal. Plantars were flexor and sensory examination was normal. On walking, he had a tilt to the left side of about 70 degrees with a normal stance and gait with reduced arm swing on the left side. The tilt of the trunk disappeared in a recumbent position. Given the rapidly progressive extrapyramidal symptoms and autonomic symptoms , the possibility of prion disease, autoimmune disorders, drug-induced and toxic disorders, and paraneoplastic etiologies were considered. He was investigated in detail . MRI of the Brain and Spinewas normal. All his hematological and biochemical tests including hemogram, fasting sugar, renal and liver functions, electrolytes and thyroid function were normal. Serology for human immunodeficiency virus, hepatitis B and hepatitis C were negative. The paraneoplastic panel came negative. A strong possibility of autoimmune disease with predominant extrapyramidal features was considered .A detailed autoimmune serological evaluation including IgLON5 antibodies was done which a showed strong positivity for antibodies to IgLON5. IgLON5 analysis was done in 1:10 serum dilution in transfected cells (Euroimmune) and came strongly positive (3 + on semi-quantitative visual grading on intensity of fluorescence) as shown in Fig. 1. Antibody titres were not done as this facility was not available at our centre. Polysomnography was normal. He had normal scores on Addenbrookes test and frontal assessment battery .

## 3 Outcome and follow-up

The patient was treated initially with a short dose of Methylprednisolone for 5 days. This was followed immediately with rituximab 1gm, given two weeks apart . At one month follow up, the patient reported a 50% improvement with respect to reduced sweating and choreiform movements. Rituximab 500 mg was repeated every 6 months .At 2 year followup , he had significant improvement of symptoms . Head control was better with significant reduction in diaphoresis.There was improvement in speed of gait with improvement in lateral tilt of the trunk. Orolingual dyskinesias and limb chorea persisted inspite of treatment.

## 4 Discussion

Here we have described an unusual manifestation of IgLON5 antibody mediated disease presenting as PS. PS or pleurothotonus is a reversible lateral flexion of the trunk that can be diminished by passive movement or supine positioning. Though Pisa syndrome was first described in Ekbom et al. [4] in association with neuroleptics use, various other causes have also been identified (supplementary table 1) [5]. Though the lateral tilt was one of the prominent presentations, he also had additional findings like chorea, head drop and autonomic dysfunction suggestive of a Pisa plus syndrome. Anti-IgLON5 disease is a relatively new autoimmune neurological disorder, first reported in 2014, by Sabater et al. [6] IgLON5 is a cell adhesion molecule associated with maintenance of the blood-brain barrier and is involved in neuroplasticity and neuroregeneration. Anti-IgLON5 disease is a tauopathy with neurodegeneration. The classic movement disorder reported is a PSP like syndrome. Sleep disturbances is a very characteristic feature and includes REM behavioural disturbances (RBD), obstructive sleep apnea, stridor, insomnia, and excessive daytime sleepiness. The spectrum of clinical abnormalities is expanding. Ganapule et al. [7] has 3 (2025) has reported five cases of frontal executive type dementia in anti-IgLON5 disease. Cases can also mimic Diffuse Lewy Body dementia (DLBD) and multiple system atrophy (MSA). Chorea involving face, trunk and limbs can be a prominent manifestation. Muscle weakness, fasciculations, myokymias and myorrhythmias and an amyotrophic lateral sclerosis (ALS)-like presentation can be a part of the disease. Oculomotor abnormalities like impaired horizontal and vertical gaze palsies, nystagmus and ptosis and gait abnormalities are also reported. Dysautonomic features may also be present in the form of urinary disturbances, constipation, and sweating abnormalities. PS is a rare manifestation of anti-IgLON5 disease. Literature review revealed three similar cases of PS in anti-IgLON5 disease (Table 1). Our patient shared a few clinical features with previously reported cases; including head drop, sweating abnormalities, choreiform movements, and gait disturbances. However, he did not exhibit sleep disturbances or cognitive impairments. Notably, he showed a good response to rituximab, unlike some of the cases described in earlier reports. Anti IgLon 5 disease is characterised by predominance of IgG4 antibodies and this may explain the good response to B cell depleting drugs like rituximab. Functional imaging of the brain, such as a PET scan, could not be performed due to financial constraints.

**Table 1:** Comparison of the clinical features and treatment of different cases reported and their response to rituximab

S. N.	Study	Age	Gender	Symptoms	Duration	Associated symptoms	IgLON5 positive	Treatment	Response to rituximab
1	Peeters et al. [1]	65	Female	Head drops and Latero-flexion of trunk	3 months	Insomnia, fatigue, dysarthria, dysphagia, falls, cognitive decline, autonomic dysfunction	Serum	Steroid, plasmapheresis and Rituximab	No response
2.	Soman Pillai et al. [2]	49	Female	Intermittent head drops, and lateral flexion of trunk	10 months	Insomnia, chorea, falls, dementia.	Serum and CSF	Steroids and IV Ig	Partial
3.	Kausthubh *et al.* [3]	58	Male	Head drop, neck and trunk dystonia to left	18 months	Sleep orthopnea, dysarthria, hypophonia, weakness of limbs	Serum, Cererospinal fluid	Steroid and Rituximab	Significant improvement
4.	Present study 2025	55	Male	Head drop, lateral flexion	1 month	Autonomic dysfunction (diaphoresis), choreiform movements, falls	Serum	Steroids and Rituximab	Significant improvement

## 5 Conclusion

Pisa syndrome can be a manifestation of Anti-IgLON5 disease. Awareness of this etiology is important as treatment with immunomodulators like rituximab helps to ameliorate symptoms.

Key takeaway 1. Anti- IgLON5 disease should be considered in any case of new onset lateral tilt of the body. 2. The clinical spectrum of anti-IgLON5 disease is expanding. 3. Presence of head drop, extrapyramidal features and autonomic dysfunction like sweating abnormalities may be point to a diagnosis of IgLON5 antibody disease. 4. Classical features of anti-IgLON5 disease like sleep disturbances and cognitive deficits may not always be present. 5. A high index of suspicion is needed for an early and correct diagnosis of anti-IgLON5 disease as it is potentially treatable with immunomodulators like rituximab.

## 6 Declaration

The authors declare that this case report represents original work and has not been submitted or published elsewhere. All authors have read and approved the final manuscript. During the preparation of this work the author(s) used ChatGPT in order to rephrase the paragraphs and formatting. After using this tool/service, the author(s) reviewed and edited the content as needed and take(s) full responsibility for the content of the published article

## 7 Ethical compliance statement

We confirm that we have read the Journal’s position on issues involved in ethical publication and affirm that this report is consistent with those guidelines. This manuscript is based on original work and has not been published whole or in part in any print or electronic media or is under consideration of publication in any print or electronic media.

## Declaration of Generative AI and AI-assisted technologies in the writing process

During the preparation of this work the author(s) used ChatGPT (3.5) to refine language and grammar as a writing tool. After using this tool/service, the author(s) reviewed and edited the content as needed and take(s) full responsibility for the content of the publication.

## Authors contributions

All authors meet the required criteria for authorship.

## CRediT authorship contribution statement

**Sindhu V. Nambiar:** Writing – review & editing, Writing – original draft, Visualization, Methodology, Investigation, Conceptualization. **Thomas Mathew:** Writing – review & editing, Supervision, Project administration. **Vishal Chandra Sharma:** Writing – review & editing, Resources, Data curation. **Arjun Jayaprakash:** Writing – review & editing, Resources. **Shagun Bhardwaj:** Writing – review & editing, Methodology, Conceptualization. **Anita Mahadevanan:** Writing – review & editing, Resources.

## Funding

No funding or financial support was received for this work.

## Declaration of competing interest

The authors declare that they have no known competing financial interests or personal relationships that could have appeared to influence the work reported in this paper.
