# Supplementary material for: An unusual case of Pisa syndrome secondary to anti-IgLon 5 disease
Source: Clin Park Relat Disord. 2026 Feb 19;14:100428. doi: 10.1016/j.prdoa.2026.100428 (PMC13080482; doi:10.1016/j.prdoa.2026.100428)
Supplement: Supplementary data 1 [file mmc1.docx]

**Table 1 (supplementary):**  Causes of Pisa Syndrome

| S. N. | Causes of Pisa Syndrome |
| --- | --- |
| 1 | Drugs: Neuroleptics, dopaminergic drugs, valproate, lithium, anticholinesterases, antiemetics |
| 2 | Alzheimers disease, Parkinsons disease, Diffuse Lewy body disease, Progressive supranuclear palsy, subacute sclerosing panencephalitis |
| 3 | Subdural hematoma |
| 4 | Normal pressure hydrocephalus |
| 5 | Late complications of pallidotomy |
| 6 | Idiopathic |
